# An Ethical Defense of a Mandated Choice Consent Procedure for Deceased Organ Donation

**DOI:** 10.1007/s41649-022-00206-5

**Published:** 2022-04-29

**Authors:** Xavier Symons, Billy Poulden

**Affiliations:** 1 Plunkett Centre for Ethics, Australian Catholic University, Sydney, NSW Australia; 2 The University of Notre Dame Australia, Fremantle, Australia

**Keywords:** Organ donation, Consent, Opt-in system, Opt-out system, Mandated choice, Autonomy

## Abstract

Organ transplant shortages are ubiquitous in healthcare systems around the world. In response, several commentators have argued for the adoption of an opt-out policy for organ transplantation, whereby individuals would by default be registered as organ donors unless they informed authorities of their desire to opt-out. This may potentially lead to an increase in donation rates. An opt-out system, however, presumes consent even when it is evident that a significant minority are resistant to organ donation. In this article, we defend a mandated choice framework for consent to deceased organ donation. A mandated choice framework, coupled with good public education, would likely increase donation rates. More importantly, however, a mandated choice framework would respect the autonomous preferences of people who do not wish to donate. We focus in particular on the Australian healthcare context, and consider how a mandated choice system could function as an ethical means to increase the organ donation rate in Australia. We make the novel proposal that all individuals who vote at an Australian federal election be required to state their organ donation preferences when voting.

## Introduction

Each year, several hundred organ donors across Australia provide organ transplants for the country’s healthcare system via the national organ donor program DonateLife. Currently, there are roughly 1700 people on the waitlist for organs in Australia, with another 12,000 people on dialysis who would benefit considerably from a kidney or liver donation (Organ and Tissue Authority n.d.). There were 110 active patients on the lung transplant waiting list and 77 active patients on the heart transplant waiting list as of 1st February 2022 (ANZOD Registry n.d.). Australia, however, has a relatively low organ donation rate compared to many other OECD nations; it is ranked 19th with 20.7 deceased people donating per million of the population. This is less than half the donation rate in Spain, which sits in first with 46.9 people per million (Arshad et al. 2019).^1^ Australia’s current organ donation policies are not sufficient to meet demand within state and territory healthcare systems. As the federal Department of Health (2021) noted on its website recently, “our donation rate has been rising, but we still need to do more.”

This paper argues that a mandated choice framework for organ donation would be an ethical way to increase the pool of vital organs available for transplant. Mandated choice is an approach to public policy questions in which people are required to make a choice about whether they will engage in a particular action. In the context of organ donation, a mandated choice policy would require individuals to make an advance decision as to whether or not they wish to donate their organs in the event of their death. Specifically, we make the novel proposal that all individuals who vote at an Australian federal election be required to state their organ donation preferences at time of voting. A mandated choice framework of this kind would be more effective than an opt-in system as it would require citizens to consider whether or not they will become organ donors whereas an opt-in system leaves this up to personal initiative. A mandated choice system, furthermore, is more ethical than an opt-out framework as it does not rest on a problematic assumption of ‘presumed consent'.

Section one of this paper offers an overview of current consent procedures for organ donation and describes the ethical context of organ donation. Section two discusses the ethics of opt-in, quasi-opt-in, and opt-out frameworks for organ donation. Section three explores how a mandated choice system might be enacted in the Australian context and the benefits that it would have. The final section of this paper critically evaluates some objections to our thesis.

## Overview

### Three Frameworks for Organ Donation

The three frameworks for consent to deceased organ donation are an opt-in system, an opt-out system, and a mandated choice system. The WHO describes an opt-in system, or explicit consent system, as a system in which cells, tissues, or organs may be removed from a deceased person if the person had expressly consented to such removal during his or her lifetime (World Health Organization 2009). An opt-out or presumed consent system is one that permits material to be removed from the body of a deceased person for transplantation unless the person had expressed his or her opposition before death (Arshad et al. 2019). A mandated choice system *is one in which* each individual is required to decide in advance if they wish to donate their organs in the event of their death.

Australia’s organ donation system has sometimes been described as an opt-in system (Organ and Tissue Authority n.d.; Bhatia and Tibballs 2018); it is more accurately described, however, as a quasi-opt-in system. In Australia, eligible persons can register their consent to organ donation with the Australian Organ Donor Register. Despite this, when a registered donor dies, their family or next-of-kin must also be consulted and give consent for organs and tissues to be donated (Organ and Tissue Authority n.d.). Families also have the right to choose for their loved ones to become donors after their death even if they are not registered (so long as, according to the family, the individual in question did not actively object to organ donation in their lifetime) (Organ and Tissue Authority n.d.). The system involves a two-step process, then, and would most accurately be described as a quasi-opt-in system.^2^

### Ethical Context

The practice of organ donation is built on the pillar of altruism—the idea that one ought to act in the interests of others even when this involves a cost for oneself. That is to say, voluntary, non-commercial organ donation is, in its essence, a beneficent and altruistic act. One person freely chooses to donate their organs to others when she or he dies. The donor’s motive is to benefit others; they do not stand to profit in any other way from donating their organs (for a detailed analysis of the psychology of altruistic organ donation, see Ferguson 2022).^3^

The preferences of patients to donate, however, may at times be unclear. This is particularly the case when patients are in an unconscious state and nearing death or when patients have just died. Clinicians may need to rely on family members to determine whether someone would have wished for their organs to be procured for transplant. Sometimes families may not know if their deceased loved one wished to become an organ donor and may instead have to take a guess as to an individual’s preferences (Neate et al. 2015). In addition, clinicians must also take into account the views of families themselves who may in some cases disagree with their deceased loved one’s decision to donate their organs. Perhaps more common, families lack sufficient time to prepare to let go of a loved one or accept that they have actually died (this often takes more time than is available when considering organ procurement in an ICU).

## The Ethics of Consent to Organ Donation

### The Ethics of Opt-In and Quasi-Opt-In Systems

It is appropriate to explore the ethical challenges associated with an opt-in system, whereby individuals are required to actively register their desire to donate if they wish to become organ donors. An opt-in system is often thought to be the most ethical system as it ensures that one’s organs are not procured without one’s explicit consent. Opt-in systems also support the beneficent and altruistic nature of organ donation, as people are actively seeking out the opportunity to benefit others while obtaining no benefit for themselves.

Our primary concern with an opt-in system is that it does not represent the personal beliefs and views of the population in its entirety. Research conducted by Donate Life Australia has found that 69% of Australians believe that becoming a registered donor is important, while only 1 in 3 Australians are registered to donate (Organ and Tissue Authority n.d.). Part of the problem would appear to be that people who may want to donate are nevertheless affected by a tendency to inertia, or a “predictable bias towards choosing options that require the least effort” (Dalal 2015). In Australia, eligible persons need to contact the Australian Organ Donor Register to register as an organ donor. This requires effort, and registration for organ donation would not seem to be a high priority for most Australians. Many people may not be willing to go out of their way to register to donate. Alternatively, people may not want to confront the question of organ donation because the topic is distasteful or because they do not know who their organs will go to. Evidence suggests that these factors influence the opinions of people who are not registered to be organ donors (Irving et al. 2012a).

Replacing an opt-in system with a mandated choice system would help address these issues, as people would be required to confront the question of organ donation and register their preferences (Beraldo and Karpus 2021). A mandated choice system compensates for biases such as a bias towards options that require less effort or a squeamishness or distaste that may lead people to avoid the question of organ donation. On a mandated choice framework, people still have a choice about whether they will donate, but they are nevertheless required to make a choice. What we are arguing for is that people ought to be required to confront the question of organ donation and make a decision about whether they will donate.

Ethical challenges also arise in systems where families are asked to make a decision about organ donation when a deceased loved one has not indicated their donation preferences (Etheredge 2021; Tennakore et al. 2021). Such a procedure is in place in most countries, opt-in system or otherwise (Young 2012). First and foremost, a family may not know what a deceased individual’s preferences would have been; they may need to engage in guesswork based on the individual’s values (Neate et al. 2015). Second, if a family has philosophical or religious reservations about donation, this consideration may trump any attempt to assess what a deceased individual would have themselves wanted. In the case of Australian indigenous communities, for example, cultural barriers can sometimes be a significant obstacle to proxy consent to organ donation for a family member (Cairnes et al. 2021).

A mandated choice system would at least limit the need for guess work on the part of families. It would make it much clearer whether someone had an enduring preference to donate their organs. Having a clear record of a patient’s preferences may also lead families to balance a consideration of their own philosophical or religious views on donation with a concern to honor the preferences of the deceased patient. Importantly, research into community attitudes to deceased organ donation found that some community members were unhappy with a system in which family members could overrule the donation decision (Irving et al. 2012b). Perhaps complementing a family veto option with a mandated choice framework may address some of these concerns, as it would ‘temper', so to speak, family decisions vis-a-vis organ donation.

### The Ethics of Opt-Out Organ Donation

As the need for organ transplants increases globally, many countries have adopted an opt-out approach or are actively considering adopting such a system to meet demand (Cook 2019; Symons 2018; Arshad et al. 2019). This may be one reason why Spain has the highest rate of deceased organ donation in the world, followed by other opt-out countries like Portugal and Belgium (English et al. 2019). Some scholars, however, argue that there is limited evidence to suggest that opt-out systems are responsible for increased organ donation rates (Abadie and Gay 2006; Rithalia et al. 2009; Rodríguez-Arias et al. 2010; Matesanz et al. 2011; Matesanz and Domínguez-Gil 2019). In any case, while there is a need to address the demand for organ donation in the health system of Australia and many other countries, we argue that an opt-out system is by no means an ethical solution to this problem. A utilitarian theorist may view the opt-out system as a significant ethical success, as the positives of the system are more availability of organs while the consequences are seemingly minimal (Boyce 2013). They might also speculate that any diminution in autonomy arising from an opt-out system—such as the fact that some citizens may not even know that such an opt-out system exists—is vastly outweighed by the benefits of an increased pool of vital organs for transplant. This system fails, however, to satisfy the requirements of respect for autonomy, undermines the dignity of deceased persons, and detracts from the fundamentally altruistic character of organ donation. Like several other theorists, we believe that the act of presumed consent takes away from the altruistic nature of the donation, as it is not at all clear that presumed consent is equivalent to an informed decision on the part of the donor. Opt-out systems are often referred to as ‘presumed consent’ systems. McEwen notes that presumed consent is a term traditionally used in health care settings where the individual is deemed incompetent or unable to make informed choices, thus allowing health care professionals to make choices that are in the best interest of the patient (McEwen 2005).^4^ He argues that adopting a presumed consent model in the setting of deceased organ donation overrides the basic right of autonomy, and ignores the fact that living people cannot make informed decisions about what happens after they die. He also argues that social intolerance to this type of paternalistic determination can manifest in resentment, mistrust with the healthcare system, and potentially even refusal to donate. Indeed, Brazil introduced a system of presumed consent to organ donation in 1997, only to abolish it a year later. One reason why this happened is thought to have been people’s distrust in the opt-out organ procurement system (Csillag 1998).

It should also be noted that family override occurs in opt-out systems also. That is to say, families can refuse donation even if a person has not opted-out. While this may be good for deceased individuals who would have otherwise opted-out, it may frustrate the preferences of deceased individuals who wanted to donate.

## A Mandated Choice System

### How a Mandated Choice System Would Work

A mandated choice system would require all people with the capacity to consent to make an informed decision about what they would like to happen to their organs in the event of their death. Organ donation consent would either be integrated into a pre-existing administrative procedure (such as driver registration or registering for a social security number), or would be a stand-alone requirement for citizens. In our view, citizens should also be required, with appropriate frequency, to reconsider their status as a potential organ donor. That is to say, citizens should be required to revisit their organ donation preferences at regular intervals throughout their lives, as their donor preferences might change with time. Health authorities, furthermore, should fund extensive public education programs so that people can make a truly informed decision about becoming an organ donor.

To be clear, a mandated choice is different from a nudged choice. ‘Nudging’ involves attempts to indirectly or subconsciously influence the kinds of options that people choose in a decision situation. We are not concerned with this. Rather, we are concerned with requiring that people make a choice, whatever their decision might eventually be. We would advocate public education about organ donation, but this is different from attempts to indirectly or subconsciously influence the behavior of social agents (for a helpful discussion of the ethics of nudging, see Schmidt and Engelen 2020).

It is necessary to have a practical means of allowing people to register and also revisit their decision to donate on a sufficiently regular basis. With this in mind—and with a focus on the Australian context—we propose that the organ donor registry be integrated into the Australian federal voting system. On our proposed model, all people would register a decision on whether or not they wish to be an organ donor when they register to vote after turning 18. The Australian Electoral Commission Roll would thus include data on voter preferences vis-a-vis organ donation. This would replace the Australian Organ Donor Register (the current register for vital organ donation in Australia). We also propose that one’s decision to donate should be revisited each time an individual votes, so that any changes in their decision be recorded on a regular basis. This could occur roughly every 3 years during an Australian federal election. Such a system would not impose any undue or additional burdens on citizens, but would still allow for a regular reconsideration of one’s decision about being a donor, and would give a relatively current picture of one’s preferences with respect to organ donation.

For this system to be effective, there would need to be a consequence for those who abstain from registering their decision to donate. Without this, there is the risk that some may ignore or avoid answering the question, thus undermining the concept behind mandated choice. We propose that there be a small financial penalty for people who do not respond to the question of organ donation status—proportionate to the penalty for failing to vote during a federal, state, or council election in Australia. Those who receive a penalty notice should have the opportunity to overturn the fine if they subsequently register their decision to donate through an online portal.

Someone might object that Australia has already trialed a mandated choice system, with limited success. Indeed, the Australian state of New South Wales (NSW) for several years had a driver registration procedure that asked registrants about their organ donation preferences. This system was discontinued in 2012 due to concerns that it was actually *impeding* consent to organ donation. Over a quarter of registrants said ‘no’ to donation when registering or renewing their driver’s license (ABC 2012). In response, we would note that the problem with the New South Wales system was not so much that people were asked about organ donation, but rather that members of the public did not know enough about the organ donation process. As one organ donation advocate noted at the time, “they’re saying no in an uneducated and uninformed way” (ABC 2012). It is important to bear in mind that the NSW mandated choice system may very well have led to more organ donation than the situation that NSW and other states currently find themselves in, where only a third of people have registered their intention to donate on the Australian Organ Donor Registry (AODR) (Moloney et al. 2022).

For a system of mandated choice to be both ethical and effective, it must be preceded by and coupled with far-reaching public education campaigns. Without this, there is a risk that organ donation rates may be reduced, much like what was seen in NSW with the driver registration procedure. We believe that improved public education campaigns about organ donation would help to remedy this issue. There is sufficient empirical evidence to suggest that public education assists in increasing organ donation rates. A 2006 American study of 490 high school students from Michigan, for example, found that a web-based intervention educating participants about the process of organ donation and organ transplantation led to a statistically significant increase in knowledge about issues related to organ donation and an increased likelihood of registering as an organ donor (Vinokur et al. 2006). Similarly, a 2010 Turkish study on the influence of an education program on organ donation rates among members of a military unit found that just one educational experience led to an increase of organ donor rates from 45.4% to 84.8% (Yilmaz 2011). We think that our proposed system—universal mandated choice plus extensive public education—will lead to an increase in donation and, in any event, will rely on a more ethical consent process.

### Benefits of a Mandated Choice System

Mandated choice would address many of the concerns about informed consent arising from both opt-out systems and quasi-opt-in systems. Individuals would make their own decision as to whether or not they wish to be organ donors. Families would not be allowed to override the decision, nor would the state be able to coerce individuals into donating. A mandated choice system, furthermore, overcomes the problem of a psychological bias against decision-making without unduly interfering with the daily lives of citizens.

A mandated choice system would almost certainly increase the number of donors, as the previously mentioned research showed most Australians believe it is important to be a donor; many just have not registered. It is also worth noting that organ donation rates have steadily increased in jurisdictions such as New Zealand, where a mandated choice policy has been in place for over three decades (Organ Donation New Zealand n.d.). In a mandated choice framework, the decision is still left in the hands of the individual, meaning that the act of becoming a donor is still in itself altruistic. For this system to be a success, there would need to be significant investment in educational programs before people make their decisions, so as not to undermine the ‘informed’ nature of the consent. Yet we believe that the cost of educating the public about Australia’s organ donation system would be a very worthwhile investment, particularly if it led to a significant increase in the number of vital organs available for transplant. Indeed, federal and state and territory governments have not hesitated to invest in organ donation in the past on account of the immense health and social benefits that come from increased donation rates.

## Objections

In this section, we will briefly explore some potential objections to the position that we have advanced. One objection that a critic might raise is that a mandated choice framework is too invasive, and that a political election is not the occasion on which to be dealing with issues pertaining to medical care and healthcare resources. One could argue that political elections should focus solely on who ought to represent an electorate. In response, we would note that healthcare policy issues regularly feature on the ballot paper of elections such as New Zealand’s recent referendum on the legalization of euthanasia; Colorado’s recent referendum on late term abortion law (Brown 2020); and the various referenda on the legalization of the sale and purchase of recreational cannabis in US states. We cannot see how requiring people to consider their status as an organ donor would be any more invasive than any of these items that have already appeared on ballot papers in liberal democratic nations. Indeed, organ donation is arguably a much less socially divisive issue than euthanasia, abortion, or recreational cannabis use.

An alternative response would be to concede the objection and instead tie organ donation to registration for a driver’s license, as is the case in New Zealand and several US states (Thaysen and Albertsen 2021). The disadvantage of this would be that fewer people would register as organ donors as fewer people opt to get a driver’s license than vote where voting is compulsory for all people over the age of 18.

A critic might also argue that a mandated choice system alone is not enough to address the various obstacles preventing people from registering as organ donors, nor would a mandated choice system stop families from overriding an individual’s desire to donate once they have died. We agree; we do not think that mandated choice is a silver-bullet. But we do believe that it will help. One reform that would complement the introduction of a mandated choice system would be improved public education about organ donation and its benefits. In addition, when healthcare professionals or lawyers are explaining and recommending advance care planning, they ought to emphasize that—because it is often enormously hard for family members to be faithful to their family member’s end of life preferences (they may even find it difficult to ‘let the person die’)—each person completing a care plan should convey the details of the plan to family members. This would assist the family in being able to ‘let go' of their loved one when the time comes. Family members are much less likely to overrule the prior decision of the person to donate organs if those conversations have been had, not just once but again and again (Bloomer et al. 2010).

Indeed, education and public awareness are in some ways even more important than a mandated choice system; we do not want our argument to be construed as an attempt to put the horse before the cart. We do, however, believe that both education and a mandated choice framework will be necessary to achieve adequate rates of organ donation in Australia to meet demand.

## Conclusion

We believe that a system of mandated choice and informed consent would be the most ethical step forward to meeting the needs of the Australian healthcare system for increased organ donation. We have argued that a mandated choice system is more effective in promoting donation than an opt-in system. Indeed, it is more in line with community views on the importance of organ donation. A mandated choice system is also more respectful of patient preferences than an opt-out system. Health authorities should seriously consider a mandated choice framework as a policy option in Australian states and territories. Further research into public education campaigns surrounding organ donation would also be of immense benefit to policy makers. In any case, policy makers ought to closely review the significant contributions to the literature in recent years that present strong evidence in favor of the adoption of a mandated choice framework for organ donation (Lin et al. 2018; Beraldo and Karpus 2021; Thaysen and Albertsen 2021).

## Data Availability

Not applicable.

## Footnotes

1 Matesanz et al. (2017) argue that Spanish organ donation rates are high due to “a specific organizational approach to ensure the systematic identification of opportunities for organ donation and their transition to actual donation and to promote public support for the donation of organs after death.”.

2 We do not deny that Australia’s quasi-opt-in system meets the legal requirements of consent. The consent procedure is not dissimilar to procedures employed with other medical and surgical interventions performed at the end of life when a patient lacks capacity.

3 Several attempts have been made to justify a reward/payment system for organ donation in the bioethics literature (Erin and Harris 2003; Rasiah et al. 2020). Yet to date, the sale and purchase of human organs remain illegal in almost all countries, with Iran being a notable exception.

4 The paradigm scenario for presumed consent is an emergency situation where (unless a patient has made representations to the contrary) doctors can presume that they are permitted to perform interventions necessary to save the patient’s life.
